# Using network analysis to personalize treatment for individuals with co-occurring restrictive eating disorders and suicidality: a proof-of-concept study

**DOI:** 10.1186/s40337-025-01259-1

**Published:** 2025-07-28

**Authors:** Lauren M. Harris, Irina A. Vanzhula, Elizabeth D. Cash, Cheri A. Levinson, April R. Smith

**Affiliations:** 1 Department of Psychological Sciences, Auburn University, 226 Thach Hall, Auburn, AL 36849-9027 USA; 2 Department of Psychological and Brain Sciences, University of Louisville, Louisville, KY USA; 3 Department of Otolaryngology – Head and Neck Surgery and Communicative Disorders, University of Louisville School of Medicine, Louisville, KY USA; 4 Brown Cancer Center, University of Louisville Health, Louisville, KY USA; 5 Division of Child and Adolescent Psychiatry and Psychology, Department of Pediatrics, University of Louisville School of Medicine, Louisville, KY USA

**Keywords:** Eating disorders, Suicide, Precision psychiatry, Idiographic, Network analysis

## Abstract

**Background:**

Nomothetic (i.e., on average) eating disorder interventions generally provide insufficient guidance for managing suicidality. The present proof-of-concept study demonstrates how idiographic network models can be used to inform a modular, highly personalized approach to treatment for individuals experiencing suicidality in the context of Anorexia Nervosa spectrum disorders (ANSD).

**Methods:**

Using 21 days of ecological momentary assessment data (105 assessment points), contemporaneous and temporal idiographic symptom networks were generated for three patients with unique clinical presentations of ANSD. For each patient, we identify the most central symptoms in their network, as well as potentially important bridge symptoms linking eating pathology and suicidality. We then provide guidelines for using this information to guide the delivery of evidence-based intervention strategies.

**Results:**

Intervention strategies may vary substantially depending upon which network statistics are used to guide treatment target selection. Bridge symptoms, or symptoms that serve as links between eating pathology and suicidality, may represent particularly promising intervention targets for individuals experiencing these conditions concurrently. Interventions which target the symptoms with the highest strength centrality may also yield symptom improvement throughout the entire network.

**Conclusions:**

Although the viability of network-informed, personalized treatment is contingent upon continued intervention development research, this approach has the potential to improve treatment outcomes for individuals with co-occurring eating disorders and suicidality.

**Supplementary Information:**

The online version contains supplementary material available at 10.1186/s40337-025-01259-1.

## Abstract (articlehighlights)

Existing treatments for eating disorders are helpful for only a minority of patients. Many individuals with eating disorders also experience thoughts of suicide, and due to the limitations of current treatments, clinicians may struggle to help patients with these conditions. Fortunately, new statistical techniques are paving the way for innovative, highly personalized approaches to treatment which allow clinicians to help patients address eating disorder symptoms and suicide-related symptoms simultaneously. This study explains how these techniques could be used to improve treatment outcomes for eating disorder patients at high risk for suicide. Although the treatment techniques described in this study hold great promise, future research is needed to ensure that they can be made accessible for clinicians and patients.

**Supplementary Information:**

The online version contains supplementary material available at 10.1186/s40337-025-01259-1.

## Introduction

Eating disorders are characterized by significantly elevated mortality risk, with the highest mortality rates occurring in Anorexia Nervosa (AN) [1]. Although medical complications are major contributors to premature death in AN [2], suicide is also a leading cause of mortality; adult females with AN are 18.1 times more likely to die by suicide than their same-age peers in the general population [3]. Atypical AN (AAN), in which all the criteria for AN are met except low body weight [4], is characterized by similar elevations in suicide risk [5, 6]. In light of growing evidence suggesting AN and AAN cannot be distinguished on the basis of clinical comorbidity or suicidality [6–8], AN and AAN are increasingly being considered manifestations of the same underlying psychopathology [9], termed here Anorexia Nervosa Spectrum Disorder (ANSD). Despite the importance of addressing the concerning rates of suicidal thoughts and behaviors in this population, there are currently no evidence-based eating disorder interventions which provide explicit guidelines for treating individuals with co-occurring ANSD and suicidality.

Evidence-based interventions for eating disorders typically adopt a nomothetic approach, resting upon the assumption that the most viable treatment targets will be similar across individuals. For example, Enhanced Cognitive Behavioral Therapy (CBT-E), which is a first-line treatment for eating disorders, is designed to target common cognitive and behavioral mechanisms observed across diagnoses, such as over-evaluation of shape and weight [10]. Given the high heterogeneity observed among individuals with eating disorders, even those sharing the same diagnosis [11], an approach developed based on averages is likely to fall short for patients who differ substantially from the modal patient for whom the treatment was designed. It is perhaps unsurprising, therefore, that CBT-E is only efficacious for approximately half of eating disorder patients [12].

Nomothetic approaches to eating disorder treatment also generally provide insufficient guidance for managing suicidal thoughts and behaviors. For example, the CBT-E manual simply recommends a “preliminary intervention” to mitigate suicide risk prior to initiating treatment [13], perhaps because suicidality among eating disorder patients is often attributed, at least in part, to comorbid conditions (e.g., depression) or personality traits (e.g., impulsivity) [14–18]. Although it remains unclear whether there is a direct link between eating disorders and suicide, it is notable that eating disorders remain significantly associated with suicidality even after accounting for comorbid disorders [18, 19], and many individuals engage in disordered eating with the explicit desire to die as a consequence of these behaviors [20, 21].The lack of clarity surrounding the origin of suicidality within eating disorders poses a dilemma for clinicians: is it best to prioritize the treatment of the eating disorder, in the hopes that a reduction in symptoms will mitigate suicide risk? Or should the clinician focus first on reducing suicidality, which may delay the treatment of eating disorder symptoms? Although it is critical to immediately intervene in the context of acute suicide risk [20], a substantial gray area remains, wherein clinicians are left to rely on their clinical judgment to determine the best course of treatment.

Improving treatment for individuals with co-occurring eating disorders and suicidality will likely require a shift away from interventions developed for the “typical” patient and towards a highly personalized (N = 1) approach [21, 22]. Although personalization of treatment is not a new idea, recent advances in idiographic methodologies [23] have paved the way for novel approaches to individualized treatment, such as those drawn from network theory [24–26]. Network theory conceptualizes mental disorders as complex, dynamic systems wherein symptoms causally interact with one another [27–29]. Network analysis, which is the quantitative application of network theory, can be used to quantify the degree and direction of interconnectedness between symptoms, as well as to identify which symptoms are most central to the maintenance of psychopathology within an individual. This information can then be used to identify the most promising treatment targets [30]. The following articles provide a useful overview of relevant concepts and terminology: Borsboom, 2017; Fried et al., 2017; Borsboom et al., 2013; Piccirillo et al., 2019 [28, 29, 31, 32].

A growing body of evidence demonstrates that network-informed, idiographic treatments are highly feasible to implement and yield clinically significant reductions in eating disorder severity across diagnoses [25]. However, this approach has not yet been applied within populations experiencing co-occurring eating disorders and suicidality. This represents a critical direction for continued research, as individuals with active suicidality represent up to 51% of eating disorder patients [32] yet are often excluded from treatment trials [33]. As an initial step towards this goal, the present proof-of-concept study demonstrates how idiographic network models can be used to inform treatment for individuals with ANSD experiencing concomitant suicidal ideation. Given the considerable heterogeneity observed across eating disorder presentations, we hypothesized that symptom networks would vary substantially across individuals, even those sharing the same diagnosis.

In the absence of universally-accepted guidelines for selecting the most viable treatment targets from longitudinal symptom data [34], we adopt the perspective that identifying and intervening on the most central symptoms in an individual’s network should yield the greatest benefit, as these symptoms are assumed to have the strongest causal influence on other symptoms [35–37]. Therefore, we focus primarily on identifying symptoms with the highest strength centrality, which represents the degree to which one symptom is connected to other symptoms within the network. To further clarify potential illness pathways between eating pathology and suicidality, we also calculate bridge centrality statistics, which may illuminate particularly important links connecting these conditions [38]. We provide guidelines for interpreting these network statistics and illustrate how clinicians may use this information to guide the delivery of evidence-based interventions which effectively address co-occurring eating pathology and suicidality. The treatment guidelines we provide are based on a modular approach to psychotherapy, wherein specific procedures from empirically-supported treatments are structured as standalone “modules” designed to target specific symptoms.

## Methods

### Procedure

Data were collected as part of an ongoing naturalistic, longitudinal study evaluating the physiological, behavioral, and psychological mechanisms linking AN symptoms and suicidality (R01MH131633). The overarching goal of this larger study is to identify potential shared risk factors for suicide-related outcomes in AN and AAN using real-time data collected via EMA and psychophysiological assessment. At baseline, participants underwent an initial evaluation to determine eligibility and assess eating disorder symptoms, suicidal ideation, suicide attempt history, and other potentially relevant symptoms (e.g., depression, emotion regulation). Eating disorder diagnoses were determined based on the eating disorder modules of the Structured Clinical Interview for DSM-5 [39], and the frequency and intensity of eating disorder behaviors and cognitions were assessed using the Eating Disorder Diagnostic Interview [40]. Body mass index (BMI) was calculated based on participants’ self-reported height and weight. The revised Self-Injurious Thoughts and Behaviors Interview (SITBI-R) was administered to evaluate the occurrence, frequency, and characteristics of suicidal ideation and suicide attempts [41].

Following their baseline evaluation, participants initiated the EMA portion of the study. All participants were required to download the m-Path mobile application [42] to their smartphone, which prompted them to complete EMA measures five times per day. To accommodate potential differences in participants’ schedules (e.g., due to shift work), participants were asked to identify a 12-h window during which they would be awake and available to complete surveys. Participants were notified to complete their first survey at the beginning of this 12-h block, and a new survey was administered every 2.5 h thereafter. Once notified that a particular survey was available, participants were given two hours to complete it. Participants completed EMA surveys for 21 days, yielding a total of 105 possible assessment points. Each survey was designed to take approximately two to four minutes to complete.

### Participants

All participants were required to be between the ages of 18 and 65, meet diagnostic criteria for AN or AAN, and report current or past suicidal ideation or a history of suicide attempts. For the purposes of the present study, we generated networks for three participants, who were chosen semi-randomly via an iterative process. First, we visually inspected contemporaneous and temporal symptom networks for all participants in the larger sample and identified a subset of participants whose networks were readily interpretable (i.e., sufficient EMA data were available to generate networks with multiple nodes and edges). From this subset of participants, we randomly selected three participants who varied with regards to age, sex/gender, and diagnosis (i.e., AN/AAN). The three participants’ ages ranged from 22 to 34, and all identified as White and non-Hispanic. Two participants self-identified as cisgender female, and one as cisgender male. Additional information regarding the clinical characteristics of these participants is provided in the Results section, but age and gender have not been matched to specific participants due to privacy considerations.

### Measures

To ensure adequate variance for network models, participants were asked to rate all assessed symptoms on a scale ranging from 0 to 100 [43].

#### Eating disorder symptoms

To evaluate eating disorder symptoms, participants responded to twenty items adapted from the Eating Disorder Examination Questionnaire [44] assessing symptoms such as restriction, binge-eating, purging, loss of control eating, compensatory behaviors, overvaluation of weight and shape, fear of weight gain, feeling fat, and body/weight-checking behaviors (e.g., weighing oneself).

#### Suicidality

To evaluate suicidality, participants responded to twelve items adapted from the revised SITBI [41] assessing passive suicidal ideation, active suicidal ideation, thoughts about a particular method, desire to live, desire to kill one’s self, suicidal intent, controllability of suicidal ideation, reasons to die, fear of death, and thoughts that suicide could be a solution to a problem they are facing.

### Data analysis

#### Statistical software

All analyses were conducted using R Statistical Software [45]. Contemporaneous and temporal networks for each participant were estimated using the *graphicalVAR* package [46]. Centrality statistics were calculated using the *qgraph* package [47], and bridge statistics were calculated using the *networktools* package [48].

#### Missing data

We ran models using only available data, without imputation. For the three participants included in the present study, the number of available EMA timepoints ranged from 45 to 96 (out of 105 possible). Table 1 provides additional information regarding the percentage of available data for each symptom, the number of timepoints at which each symptom was assessed, and the number of days on which each symptom was assessed.

**Table 1:** EMA eating disorder and suicidality items with the highest means for each patient

Patient	Symptom	Label	Item	Mean (SD)	Available %	Timepoints (Days)
1	ED	Feel fat	I feel fat	72.27 (16.56)	91.43%	96 (21)
		Fear weight	I am terrified of gaining weight	66.63 (14.95)	91.43%	96 (21)
		Worth weight	My worth as a person (how I think about myself) is influenced by my weight and/or shape	66.31 (14.18)	91.43%	96 (21)
		Skip meals urge	I had the urge to skip meals (whether or not I actually skipped meals)	63.01 (12.69)	91.43%	96 (21)
	Suicidality	Desire live	How strong is your desire to live?	50.80 (13.87)	91.43%	96 (21)
		Fear of death	I am afraid to die	31.98 (19.34)	91.43%	96 (21)
		Reasons to die	There are more reasons to die than to live	16.73 (11.05)	91.43%	96 (21)
		Passive ideation 1	Life is not worth living to me	15.31 (6.48)	91.43%	96 (21)
2	ED	Worth weight	My worth as a person (how I think about myself) is influenced by my weight and/or shape	89.49 (9.53)	71.43%	75 (21)
		Food rules restrict	I have tried to follow definite rules regarding eating in order to influence my shape or weight	80.64 (21.50)	71.43%	75 (21)
		Feel fat	I feel fat	74.40 (21.75)	71.43%	75 (21)
		Restrict food urge	I had the urge to restrict my food intake (whether or not I actually restricted my food intake)	67.64 (19.79)	71.43%	75 (21)
	Suicidality	Fear of death	I am afraid to die	68.69 (19.05)	71.43%	75 (21)
		Desire live	How strong is your desire to live?	59.16 (17.50)	71.43%	75 (21)
		Passive ideation 1	Life is not worth living to me	45.35 (17.97)	71.43%	75 (21)
		Reasons to die	There are more reasons to die than to live	36.53 (17.04)	71.43%	75 (21)
3	ED	Feel fat	I feel fat	63.38 (15.29)	42.45%	45 (20)
		Fear weight	I am terrified of gaining weight	57.40 (16.55)	42.45%	45 (20)
		Mirror checking	I checked my appearance (e.g., in the mirror, on my phone, in a picture)	55.00 (20.03)	42.45%	45 (20)
		Restrict food urge	I had the urge to restrict my food intake (whether or not I actually restricted my food intake)	52.58 (20.93)	42.45%	45 (20)
	Suicidality	Desire live	How strong is your desire to live?	44.69 (11.92)	42.45%	45 (20)
		Reasons to die	There are more reasons to die than to live	40.73 (16.14)	42.45%	45 (20)
		Passive ideation 1	Life is not worth living to me	38.56 (15.39)	42.45%	45 (20)
		Problem solving	I thought that suicide could be a way to solve the problem I was facing	37.62 (19.77)	42.45%	45 (20)

Means were calculated by averaging scores for EMA items over the 21-day data collection period

#### Symptom selection

Emerging evidence suggests that six nodes may be ideal for idiographic networks based on data with 75–100 observations [49]; however, there are currently no universally-accepted guidelines for identifying the most important nodes from large datasets of symptoms, and prior research has found that eight-node models yield useful information for treatment target selection in an eating disorder sample [37]. We made the a priori decision to include eight total symptoms in each network: four eating disorder symptoms, and four suicide-related symptoms. To select the most relevant symptoms from each domain within each individual, we identified the symptoms with the highest means in each category across all 21 days of data collection (Tables 1, 2).

**Table 2:** Most central symptoms and associated treatment options for each patient

Patient	Treatment Targets	Centrality Statistics	Evidence-Based Intervention Options	Example Treatment Plan
1	Feel fat	Contemporaneous Strength = 0.92 Temporal OutStrength = 0.39	Exposure; imaginal exposure; mindfulness	1. Introduce self-monitoring to address **urges to skip meals** 2. Identify core values and brainstorm pleasant activities to reduce **passive ideation** 3. Conduct pie chart exercise to illustrate impact of **shape/weight overvaluation** 4. Introduce exposure techniques for reducing discomfort with **feeling fat** and de-catastrophizing worst-case scenarios of **feared weight gain** 5. Provide mindfulness-based strategies for improving acceptance of **weight-related cognitions and emotions**
	Fear weight	Contemporaneous Strength = 0.83	Imaginal exposure	
	Passive ideation 1	Contemporaneous Bridge Strength = 0.49	DBT skills; Behavioral Activation	
	Skip meals urge	Contemporaneous Bridge Strength = 0.10 Temporal OutStrength = 1.10 Temporal Bridge OutStrength = 0.49	CBT-E self-monitoring	
	Worth weight	Temporal Bridge OutStrength = 0.18	CBT-E cognitive techniques; mindfulness-based acceptance techniques	
2	Food rules restrict	Contemporaneous Strength = 0.22 Contemporaneous Bridge Strength = 0.13 Temporal OutStrength = 0.85 Temporal Bridge OutStrength = 0.35	CBT-E behavioral experiments	1. Design behavioral experiments to challenge **food rules** 2. Identify core values and pleasant activities to enhance belief that life is worth living and reduce **passive ideation** 3. Generate a safety plan to mitigate suicide risk if **passive ideation** intensifies or **fear of death** decreases 4. Highlight dissonance between harmful eating disorder behaviors and **fear of death** using Socratic method
	Fear of death	Contemporaneous Strength = 0.20 Contemporaneous Bridge Strength = 0.20	Means safety; safety planning	
	Passive ideation 1	Temporal OutStrength = 0.71 Temporal Bridge OutStrength = 0.25	DBT skills, such as Accumulating Positive Emotions; Behavioral Activation	
3	Mirror checking	Contemporaneous Strength = 1.07	CBT-E cognitive strategies; mindfulness-based acceptance techniques	1. Enhance acceptance and non-judgment of thoughts and feelings about the body (e.g., **feeling fat**) using mindfulness techniques 2. Introduce self-monitoring to track factors associated with **mirror checking** 3. Demonstrate how urge surfing could be used to manage **mirror checking** and **urges to restrict** 4. Incorporate exposure techniques to reduce discomfort with **feeling fat** and anxiety associated with **feared weight gain** 5. Identify core values and pleasant activities to increase **desire to live** 6. Create a list of reasons for living to challenge thoughts about **reasons to die**
	Feel fat	Contemporaneous Strength = 0.93 Contemporaneous Bridge Strength = 0.39	Exposure therapy; imaginal exposure; mindfulness-based cognitive therapy	
	Desire live	Contemporaneous Bridge Strength = 0.64	DBT skills, such as Accumulating Positive Emptions; Behavioral Activation	
	Fear weight	Temporal OutStrength = 0.51 Temporal Bridge OutStrength = 0.30	Imaginal exposure	
	Reasons to die	Temporal OutStrength = 0.47	Reasons for living	
	Restrict food urge	Temporal Bridge OutStrength = 0.46	CBT-E self-monitoring	

Only the two symptoms with the two highest values for each centrality statistic (i.e., contemporaneous and temporal strength; contemporaneous and temporal bridge OutStrength) are listed here. In some instances, the same symptom was associated with high values on multiple centrality statistics (e.g., demonstrated both the greatest OutStrength and the greatest contemporaneous bridge strength), resulting in a smaller number of treatment targets for certain patients. Bolded text is used to highlight the symptom being addressed in the Example Treatment Plan

#### Network estimation

In network analysis, symptoms are referred to as “nodes,” and links between symptoms are referred to as “edges.” Edges can be directed (i.e., reflect the direction of a relationship between two nodes) and/or weighted (i.e., reflect the strength of the relationship between two nodes) depending on the type of network.

**Fig. 1:**
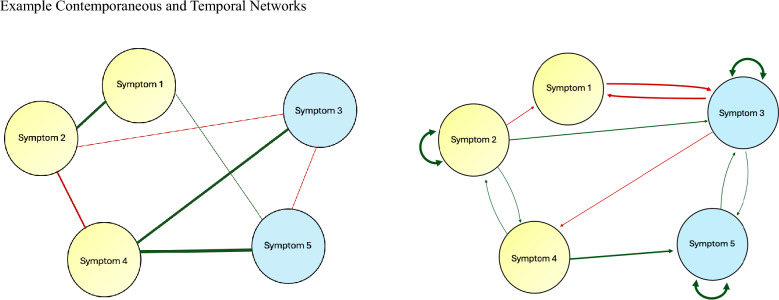
Example Contemporaneous and Temporal Networks. *Note:* In each network depicted here, symptoms (i.e., “nodes”) were drawn from two distinct communities, represented by differently colored circles (i.e., blue and yellow). Lines connecting nodes are known as “edges.” Green lines represent positive associations between symptoms, and red lines represent negative associations. The thickness of each line depicts the strength of the relationship between the two nodes it connects. The left figure depicts an example of a contemporaneous (i.e., undirected) network. The right figure depicts an example of a temporal (i.e., directed) network. Whereas the contemporaneous network depicts relationships between symptoms at a single timepoint, the temporal network depicts relationships between symptoms from one timepoint to the next, and the direction of the relationship is represented by a single-headed arrow

Figure 1 depicts simplified examples of contemporaneous and temporal networks comprising five nodes. Contemporaneous networks evaluate whether symptoms predict one another at the same timepoint. In a contemporaneous network, edges represent partial correlations between symptom variables, controlling for all other variables at the same timepoint and the previous timepoint. Contemporaneous networks are sometimes described as “undirected” networks. Temporal networks, on the other hand, are sometimes referred to as “directed” networks; they can be used to investigate whether symptoms predict one another over time. In these networks, edges with arrowheads pointing from one node to another indicate that the first node predicts the second node at the subsequent timepoint [50]. Therefore, our temporal networks illustrate how all symptoms predicted one another over the 21-day assessment period, controlling for all other symptoms at the previous assessment.

For contemporaneous networks, our primary statistic of interest was strength centrality, an overall measure of connectedness which quantifies the sum of the edge weights between a particular node and all its connecting nodes. We also calculated bridge strength for contemporaneous networks, which quantifies the connectivity between a particular node and other disorders or “communities” within the network [51]. Communities are pre-defined based on clinical criteria, not network analytic procedures, and contain symptoms that are theoretically linked to a particular disorder. For the purposes of the present study, eating disorder symptoms and suicidality-related symptoms were conceptualized as reflecting two distinct communities; therefore, the items with the highest bridge strength represent the strongest links between eating pathology and suicidality for a particular patient.

For temporal networks, we calculated two types of strength centrality: InStrength and OutStrength. InStrength (i.e., the sum of incoming path weights for a specific node) can be used to quantify which symptoms receive the most “input” *from* other symptoms in the network, whereas OutStrength (i.e., the sum of outgoing path weights for a specific node) can be used to quantify which symptoms exert the greatest influence *on* other symptoms in the network. We also calculated bridge InStrength (i.e., the sum of incoming inter-community path weights for a specific node) and bridge OutStrength (i.e., the sum of outgoing inter-community path weights for a specific node) to quantify which symptoms receive the most input from, or exert the greatest influence on, symptoms in the other community.

#### Intervention selection

The top two symptoms for each centrality statistic of interest (i.e., contemporaneous strength, temporal strength, contemporaneous bridge strength, temporal bridge OutStrength) were identified for each participant. Following the precedent set by prior studies in this domain, we strove to match each of these symptoms to an appropriate evidence-based intervention or technique theorized to target that symptom [37, 38]. In situations where evidence-based interventions for specific symptoms were not available, we drew upon the available theoretical and empirical literature to identify potentially viable intervention techniques or considered ways in which existing strategies could be modified to address new treatment targets.

## Results

**Fig. 2:**
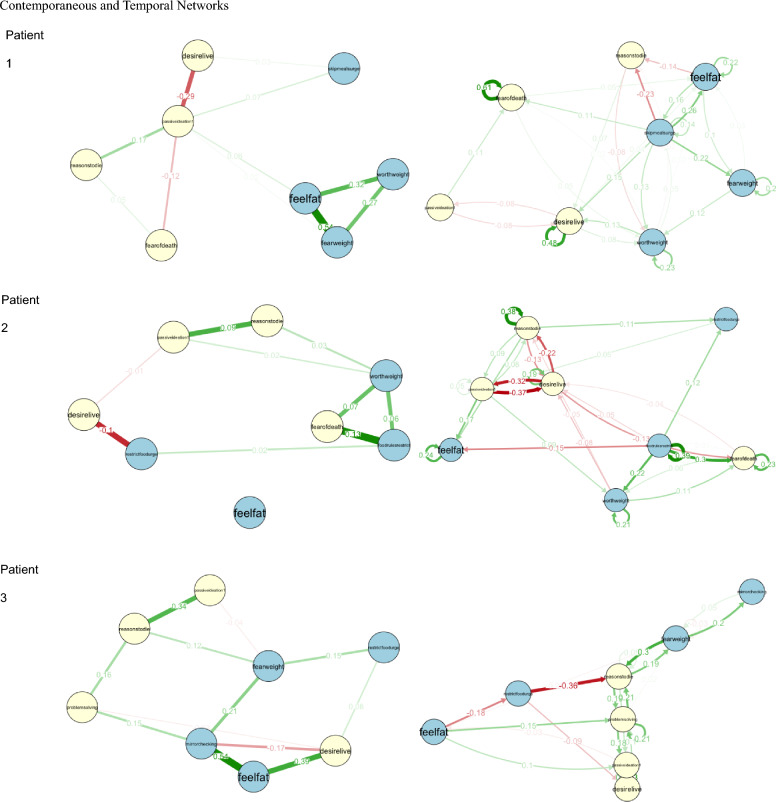
Contemporaneous and Temporal Networks. *Note* Contemporaneous networks are shown in the top row and temporal networks are below. Light yellow nodes represent suicide-related symptoms, and light blue nodes represent eating disorder symptoms. Green lines indicate positive relationships and red lines indicate negative relationships between symptoms

**Fig. 3:**
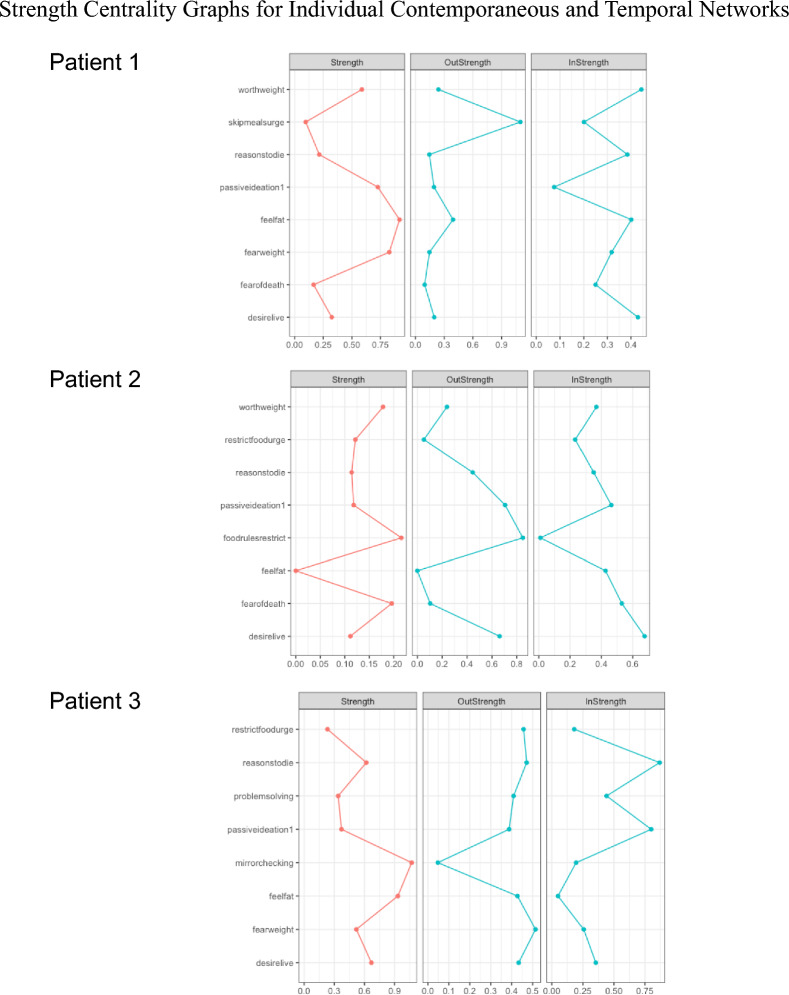
Strength Centrality Graphs for Individual Contemporaneous and Temporal Networks. *Note* Red lines represent centrality statistics from contemporaneous networks and blue lines represent centrality statistics from temporal newtorks

**Fig. 4:**
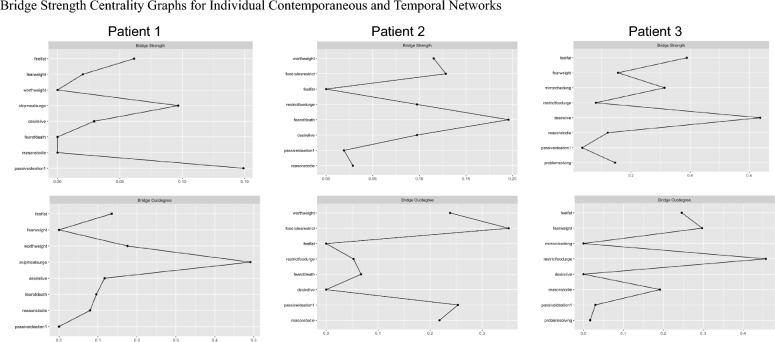
Bridge Strength Centrality Graphs for Individual Contemporaneous and Temporal Networks. *Note* Centrality statistics from contemporaneous networks are shown in the top row, and statistics from temporal networks are below

Idiographic symptom networks generated for three participants, henceforth referred to as Patients 1–3, are described below. Visualizations of each patient’s contemporaneous and temporal networks are provided in Fig. 2. Strength centrality graphs and bridge strength centrality graphs are depicted in Figs. 3 and 4, respectively. Supplementary Tables 1 and 2 provide detailed network statistics. For each patient, we discuss how information gleaned from these models may be used to guide personalized treatment which effectively targets co-occurring disordered eating and suicidality. Although these examples are not exhaustive, they are intended to illustrate how idiographic networks can facilitate modifications to treatment as usual to improve outcomes for individuals who may not represent the typical eating disorder patient.

### Patient 1

Patient 1 received a diagnosis of AAN (BMI = 22.5). At baseline, they reported a history of multiple lifetime suicide attempts, as well as current suicidal ideation. They also described recurrent engagement in several eating disorder behaviors, including subjective binge eating and fasting to control weight. For Patient 1, the eating disorder symptoms with the highest means across time (as assessed by EMA) were feeling fat, fear of weight gain, overvaluation of weight/shape, and urges to skip meals. Suicidality items with the highest means included desire to live, fear of death, reasons to die, and passive ideation. As such, these eight items were included in the network.

In Patient 1’s contemporaneous network, the items with the greatest strength centrality were feeling fat and fear of weight gain; the strongest bridge symptoms were passive suicidal ideation and the urge to skip meals. In this patient’s temporal network, the items with the greatest OutStrength were the urge to skip meals and feeling fat; the symptoms with the greatest bridge OutStrength were the urge to skip means and overvaluation of weight/shape.

#### Contemporaneous network

Based on Patient 1’s contemporaneous network, multiple approaches to treatment are possible. One option is an approach which directly targets symptom pathways between eating pathology and suicidality. This patient’s strongest contemporaneous bridge symptom was passive suicidal ideation, which was more strongly connected to eating disorder symptoms than any other suicidality symptom. Conversely, the eating disorder symptom with the strongest bridge centrality was the urge to skip meals, which was more strongly connected to suicidality symptoms than any other eating disorder symptom. If the clinician believes that the patient’s suicidality stems from their eating disorder symptoms, they may opt to begin by targeting the patient’s urge to skip meals by introducing self-monitoring, discussing the role of restriction in maintaining symptoms, and evaluating how eating patterns might be improved. Otherwise, if the clinician and patient choose to begin by addressing the patient’s passive suicidal ideation, they may begin treatment with modules intended to enhance the patient’s belief that life is worth living. Dialectical Behavior Therapy (DBT) skills such as “accumulating positive emotions,” or Behavioral Activation techniques focused on identifying and acting on core values, may be particularly well-suited to addressing this treatment aim.

The clinician may also opt to simply focus on the symptoms with the greatest strength centrality, which were feeling fat and fear of weight gain; these symptoms were most strongly linked to other symptoms in the network at the same timepoint (i.e., in the span of seconds). To target these symptoms, which are often conceptualized as facets of body image concern [13, 52, 53], the clinician might implement techniques drawn from exposure therapy [54]. For example, imaginal exposure [55] may be used to help the patient cope with their perceived “worst-case scenario” of gaining weight. Exposure may also be paired with cognitive techniques which help the patient challenge distorted cognitions regarding the consequences of changes to their body size. Alternatively, depending on the patient’s values and preferences, the clinician might opt to implement a mindfulness-based approach [56] which emphasizes acceptance and non-judgment towards thoughts and feelings about one’s body. Techniques could be drawn from interventions such as DBT (e.g., non-judgmental stance, mindfulness of current emotion) or Mindfulness-Based Cognitive Therapy (MBCT; e.g., acceptance of oneself and one’s body).

#### Temporal network

In temporal networks, the symptoms with the greatest OutStrength are hypothesized to play a key role in symptom maintenance through their influence on other symptoms. For Patient 1, feeling fat and urges to skip meals demonstrated the greatest OutStrength, indicating that intervening on these symptoms should disrupt several key symptom relationships in the patient’s network. For example, addressing the patient’s urge to skip meals should also address subsequent fear of weight gain, overvaluation of shape/weight, feeling fat, and fear of death. Notably, bridge centrality statistics indicate that the urge to skip meals was also contemporaneously linked to Patient 1’s “community” of suicidality symptoms; therefore, this represents a particularly promising treatment target for effectively and efficiently addressing co-occurring eating pathology and suicidality for Patient 1.

### Patient 2

Patient 2 received a diagnosis of AN-restricting subtype (BMI = 18.2). At baseline, they reported current and past suicidal ideation. They described frequent engagement in intense exercise but denied binge eating or purging. The eating disorder symptoms with the highest means across time for Patient 2 were overvaluation of weight/shape, following food rules, feeling fat, and urges to restrict food intake. The suicidality items with the highest average scores were fear of death, desire to live, passive ideation, and reasons to die.

The items with the greatest contemporaneous strength centrality for Patient 2 were following food rules and fear of death; these symptoms also demonstrated the greatest contemporaneous bridge strength. The items with the greatest temporal OutStrength were following food rules and passive ideation; these were also the symptoms with the highest bridge OutStrength.

#### Contemporaneous network

For Patient 2, the symptoms with the greatest contemporaneous strength centrality were the same as those with the greatest bridge strength centrality: namely, following strict food rules and fear of death. Therefore, treatment would unfold likely similarly regardless of which centrality indices were used. To reduce adherence to food rules, Patient 2 and their therapist may work together to design behavioral experiments which challenge the patient’s assumptions about the consequences of not adhering to these rules. In session, the patient may also be encouraged to reflect on how their food rules contribute to the maintenance of eating disorder symptoms.

Few interventions explicitly address fear of death, despite its theoretical importance in the transition from suicidal ideation to suicidal behavior [57]. Notably, because the EMA items used in the present study assessed *fear* of death (“I am afraid to die”) rather than *fearlessness* about death (which may increase the likelihood of a lethal suicide attempt when suicidal intent is present, this item may more accurately reflect the patient’s concerns that their eating disorder could be putting their life at risk, rather than serving as an indicator of suicide risk. A clinician working with Patient 2 may wish to explore these fears with the patient and use this as an opportunity to highlight the dissonance between the patient’s values (e.g., living a long and healthy life) and their eating disorder behaviors. Nevertheless, for Patient 2, it may be important to develop a safety plan that can be implemented when suicidal ideation arises. Doing so should have the added benefit of reducing eating disorder symptoms, as passive ideation represents the strongest bridge between eating pathology and suicidality in this patient’s contemporaneous network.

#### Temporal network

Patient 2’s temporal network indicates that the symptoms with the greatest OutStrength and bridge OutStrength are following strict food rules and passive suicidal ideation (i.e., thinking that life is not worth living). Adherence to food rules plays a key role in both the temporal and contemporaneous networks for this patient, in addition to serving as a key bridge symptom between eating disorder symptoms and suicidality; therefore, it is likely that this would represent a key treatment target for Patient 2 regardless of which network statistics were used to guide treatment. This patient’s temporal directed network and bridge centrality statistics indicate that it may also be particularly critical to address their passive suicidal ideation, which should have beneficial downstream effects on eating disorder symptoms over time due to its relatively high bridge OutStrength. Therefore, Patient 2 may benefit from interventions which strengthen their belief that life is worth living, including techniques focused on identifying and acting on core values.

### Patient 3

Patient 3 received a diagnosis of AAN (BMI = 31.1). At baseline, they reported current and past suicidal ideation, as well as a lifetime history of non-suicidal self-injury and multiple suicide attempts. They described engaging in several disordered eating behaviors over the last year, including binge eating, self-induced vomiting, fasting, and intense exercise; however, the frequency of these behaviors was not sufficient to warrant a diagnosis of Bulimia Nervosa. For Patient 3, the eating disorder symptoms with the highest means across time were feeling fat, fear of weight gain, mirror checking, and urges to restrict food intake. Suicidality items with the highest means included desire to live, reasons to die, passive ideation, and thinking about suicide as a solution to a problem.

In Patient 3’s contemporaneous network, the symptoms with the greatest strength centrality were mirror checking and feeling fat; the symptoms with the greatest bridge strength centrality were desire to live and feeling fat. In their temporal network, fear of weight gain and reasons to die demonstrated the greatest OutStrength; urges to restrict and fear of weight gain demonstrated the greatest bridge OutStrength.

#### Contemporaneous network

Bridge statistics from Patient 3’s contemporaneous network indicated that feeling fat was strongly connected to their symptoms of suicidality, suggesting that this may be an especially important early treatment target to mitigate suicide risk. The contemporaneous network for Patient 3 also indicated that feeling fat, along with mirror checking, were strongly connected to other symptoms in the network at the same timepoint (i.e., demonstrated the greatest strength centrality). To address feeling fat, interventions aimed at reducing body dissatisfaction (e.g., mindfulness techniques to enhance acceptance and non-judgment towards thoughts and feelings about the body) may be beneficial. To target mirror checking, the clinician may provide psychoeducation about how mirror checking and other body checking behaviors may serve to maintain the patient’s eating disorder symptoms and help the patient implement skills to delay or prevent mirror checking when urges arise.

#### Temporal network

Because the strongest symptoms in Patient 3’s temporal network did not overlap with the strongest symptoms in their contemporaneous network, interventions might differ substantially depending on which network was used to guide treatment. The temporal bridge network for Patient 3 indicated that urges to restrict were strongly connected to suicidality for this patient. Therefore, it may be important for treatment to include a component of self-monitoring and food logging to determine how eating patterns might be improved. The clinician and patient might also work together to identify how urges to restrict serve to maintain this patient’s eating disorder symptoms and suicidality over time. Techniques drawn from DBT, such as urge surfing, may be incorporated to help the patient tolerate and reduce urges to restrict [58].

Bridge statistics from this patient’s temporal network also indicated that reasons to die were more strongly connected to symptoms of eating pathology than any other suicidality symptom. To address reasons to die (i.e., “There are more reasons to die than to live”), the clinician may work with the patient to develop a list of reasons for living, a technique which has been shown to effectively reduce the likelihood of suicidal ideation and attempts [59].

Patient 3’s temporal network indicated that the symptoms with the greatest OutStrength were fear of weight gain and reasons to die, suggesting that these symptoms influenced the maintenance of other symptoms in the network over time. In addition to the interventions described above, the clinician might draw upon exposure techniques (e.g., imaginal exposure) to target fear of weight gain by helping the patient habituate to the anxiety produced by the thought of gaining weight, as well as to facilitate coping with the potential consequences of weight gain [55].

## Discussion

Using three unique patients as case examples, the present proof-of-concept study demonstrated how various network statistics could be used to guide intervention planning and treatment target selection for individuals with co-occurring ANSD and suicidality. Our findings highlight that personalized treatment approaches are likely to differ substantially depending on which network statistics are used, even within the same individual. Interventions that prioritize addressing pathways between eating pathology and suicidality over time, for example, may differ from those which primarily aim to target symptoms with strong contemporaneous connections to other symptoms in the network.

Because standardized guidelines for implementing treatment based on idiographic network models do not yet exist, there are several practical issues that must be addressed before best practice recommendations can be established. These issues have been reviewed previously [26, 33, 37], and many questions remain unanswered, including: (1) which symptoms to assess, and how often; (2) which items to include in idiographic models; (3) which network statistics to use to identify treatment targets; and (4) how many symptoms to target, and in what order. Many of these issues become even more complex when attempting to address multiple comorbid conditions. For example, the question of treatment order is of particular importance for co-occurring eating disorders and suicidality; if there are differential effects of treating one condition before the other, this ordering effect could have serious implications for patient safety. Comorbidity also complicates the question of what symptoms to include in idiographic networks; in addition to core symptoms from each condition of interest, it may be necessary to consider the influence of variables such as psychological wellbeing, personality traits, or interpersonal functioning to fully understand how psychopathology is maintained. However, because current guidelines recommend generating networks with a small number of nodes (e.g., approximately six nodes when 75–100 observations are available) to ensure clinical feasibility [49], it is not always possible to include all potentially relevant symptoms. In the present study, we therefore opted to prioritize including only symptoms which directly tapped into eating disorder psychopathology and suicidality. Clearly, substantial future research is needed to identify which strategies for symptom assessment, target selection, and intervention yield the best clinical outcomes.

As others have noted [37], we also emphasize that most clinicians do not have the time or resources necessary to develop expertise in network analysis. Our field’s ability to disseminate and implement highly personalized interventions will depend upon collaborations between experts in multiple fields to ensure that clinicians have access to cost-effective, accurate, and reliable systems for interpreting network models and guiding treatment based on these data; for example, usage of a clinician-friendly software program that automatically calculates treatment targets based on network algorithms [60].

In addition to clarifying optimal strategies for implementing network-based personalized treatments more broadly, there are several issues regarding the treatment of ANSD and suicidality in particular which warrant further consideration. First, existing interventions for AN (e.g., CBT-E) have garnered, at best, modest research support [61], and there are no empirically-supported treatments for AAN [62]. There is also currently no “gold-standard” intervention for suicidality; on average, treatments reduce the likelihood of self-injurious thoughts and behaviors by less than 10% [63]. Although the intention of idiographic interventions is to circumvent the need for nomothetic “one-size-fits-all” approaches, personalization of treatment nevertheless requires identifying evidence-based strategies and techniques that can be matched to an individual’s specific symptoms. In the absence of a compelling research base, it is challenging to predict which elements of existing treatments should be retained as viable intervention strategies [64], and to which symptoms they should be matched. Furthermore, because most existing interventions are based on diagnostic categories, rather than specific symptoms, there are also several key symptoms for which evidence-based interventions do not yet exist [37]. The viability of network-informed personalized treatment is contingent upon continued intervention development research; to improve treatment outcomes for individuals with heterogeneous presentations, it will be critical to ensure that evidence-based strategies are available for all potentially relevant symptoms.

### Clinical implications

Despite these caveats, there are nevertheless several promising paths forward. Pilot studies evaluating the efficacy of network-informed eating disorder interventions have primarily implemented techniques drawn from CBT and third-wave treatments, and results demonstrate that these approaches yield significant reductions in several symptoms often present in ANSD, including dietary restriction, body dissatisfaction, and cognitive restraint [38]. Although randomized controlled trials are underway to determine whether network-informed personalized interventions can outperform established eating disorder treatments such as CBT-E [65], these preliminary results underscore that many of the strategies used in mainstream treatments can be highly efficacious, especially when appropriately matched to an individual patient’s needs. The modest efficacy of established eating disorder interventions is almost certainly not a consequence of ineffective strategies; rather, these interventions may simply provide insufficient opportunities for personalization, resulting in worse outcomes for individuals who do not represent the modal patient for whom the treatment was developed. Combining a network analytic approach to treatment target selection (i.e., identifying the most viable treatment targets), with a modular approach to treatment (i.e., implementing only the most relevant intervention components, in the appropriate order) has the potential to greatly improve outcomes for individuals with heterogenous presentations of eating pathology, and does not necessitate a complete overhaul of established evidence-based interventions.

Existing evidence-based interventions for suicidality are also amenable to further personalization. Indeed, in light of increasing evidence demonstrating the limited effectiveness of current treatments for suicidal thoughts and behaviors [63], experts have called for a paradigm shift in suicide prevention which routes patients to the optimal treatment for their current suicidal state [66]. This type of flexible approach is especially well-suited to interventions which can be delivered in a modular format, as it may not be feasible (or necessary) for suicidal individuals to undergo the entirety of an evidence-based treatment protocol in situations of increased risk. Some interventions for suicidality, such as DBT, have the advantage of already being modularized [67]. Single-session interventions, such as the Collaborative Assessment and Management of Suicidality Brief Intervention (CAMS-BI), could also be delivered as standalone treatment modules to target relevant suicide-related symptoms, such as motivation to live [68]. Drawing from these modules, a clinician can easily select and implement key strategies and procedures depending on a patient’s current level of risk or, as we have demonstrated, using idiographic networks created with EMA data collected during initial stages of treatment.

In addition to enhancing flexibility and personalization, network-informed, modular approaches to treating suicidality offer an alternative to highly resource-intensive interventions, which are not accessible for many individuals due to barriers such as cost and provider availability [66]. Rather than referring a suicidal eating disorder patient to a year-long comprehensive DBT program, for example, it may be sufficient for a clinician to briefly implement specific DBT strategies targeting their most central symptoms of suicidality, then turn the focus back to eating disorder symptoms in subsequent sessions. Although this approach still requires the clinician to be thoroughly trained in relevant intervention techniques, it can be accomplished using substantially fewer resources, and likely at a much lower cost to the patient. Preliminary evidence suggests that implementation of brief modular approaches to target suicide-related symptoms are acceptable and feasible additions to outpatient CBT [69], though much additional research is needed to evaluate the effectiveness of implementing suicide intervention modules in the context of a completely idiographic intervention.

Notably, developing network-based interventions requires patients to disclose sensitive information regarding their symptoms, typically before treatment even begins, to ensure identification of the most relevant treatment targets. However, many individuals may be unwilling to disclose suicidality to their providers, perhaps for fear of involuntary hospitalization, or concerns about others (e.g., family members) finding out [70]. These concerns might be particularly salient for individuals with ANSD, who may have had previous negative experiences with hospitalization or family involvement in treatment [71]. As methods for developing personalized treatments for co-occurring eating disorders and suicidality are refined, it will be crucial to ensure that patients feel comfortable accurately and honestly reporting their symptoms to ensure that treatment is appropriately tailored to their needs.

### Future directions

In addition to the issues described above, several additional topics warrant continued consideration in future research. Although the present study centered on network-informed approaches to treating concomitant ANSD and suicidality, it stands to reason that a similar approach could be implemented to address suicidality in the context of any other eating disorder. Network-informed approaches are inherently transdiagnostic because they do not require the clinician to provide treatment based on the presence or absence of a categorical diagnosis; rather, relevant symptoms are assessed and targeted individually. This symptom-centric approach ensures that treatment is tailored to each patient’s unique presentation, rather than assuming that the same small number of symptoms will be equally important for everyone sharing the same diagnosis. Consequently, network-informed interventions are also highly amenable to fluctuating diagnostic systems, including both categorical and dimensional classification models. Future research should continue to explore the feasibility of these approaches across the spectrum of eating pathology, particularly as our understanding of eating disorders continues to evolve [72].

Additional research is also necessary to determine how to best conceptualize suicidality in the context of eating pathology. In the present study, we treated eating disorder symptoms and suicidality as two distinct “communities,” as this allowed us to identify putative illness pathways between eating pathology and suicide for each patient. This approach is consistent with prior network analytic approaches to modeling psychiatric comorbidity [73, 74]. However, conceptualizing an eating disorder and suicidality as separate conditions may not accurately reflect the relationship between these phenomena. For example, consider that in our current diagnostic system, suicidality is not considered a “symptom” of AN, yet the prevalence of suicidal ideation in AN approaches 50% [34]. In contrast, the prevalence of suicidal ideation in adults with Major Depressive Disorder (MDD) is approximately 40%, yet suicidality is considered a symptom of MDD [4]. For some individuals, it may be more accurate to consider suicidality a symptom of the eating disorder, rather than a separate phenomenon. Indeed, prior studies have found that reductions in eating pathology mediate changes in suicidal ideation during treatment [75], suggesting that suicidality is closely linked to the severity of eating pathology. Additional research is needed to continue to clarify the mechanisms that produce and maintain suicidality among individuals with eating disorders, and the extent to which individual differences influence the covariation of these conditions; doing so will inform how these symptoms can be best incorporated into idiographic symptom networks.

## Conclusions

The present proof-of-concept study illustrated how network-informed, personalized treatment might be implemented for individuals with typical and atypical AN at elevated risk for suicide. Although this approach holds great promise for the development of targeted interventions for co-occurring eating disorders and suicidality, substantial additional research is needed to evaluate its feasibility and efficacy. We look forward to continued research which further refines the strategies used to develop, implement, and disseminate evidence-based personalized treatments for individuals experiencing a wide range of psychopathology.

## Supplementary Information

Additional file 1.

## Data Availability

The datasets generated or analyzed during this study are available from the corresponding author on reasonable request.

## Abbreviations

AN: Anorexia nervosa
AAN: Atypical anorexia nervosa
ANSD: Anorexia nervosa spectrum disorders
CAMS-BI: Collaborative assessment and management of suicidality brief intervention
CBT: Cognitive behavioral therapy
CBT-E: Enhanced cognitive behavioral therapy
DBT: Dialectical behavior therapy
EMA: Ecological momentary assessment
MBCT: Mindfulness-based cognitive therapy
MDD: Major depressive disorder
SITBI: Self-injurious thoughts and behaviors interview
