# Supplementary material for: Using network analysis to personalize treatment for individuals with co-occurring restrictive eating disorders and suicidality: a proof-of-concept study
Source: J Eat Disord. 2025 Jul 28;13:156. doi: 10.1186/s40337-025-01259-1 (PMC12306141; doi:10.1186/s40337-025-01259-1)
Supplement: Supplementary file 1 — Additional file 1. [file 40337_2025_1259_MOESM1_ESM.docx]

**Supplementary Table 1**

Strength Centrality for Contemporaneous and Temporal Networks

| Patient | Network | Node | Strength | InStrength | OutStrength |
| --- | --- | --- | --- | --- | --- |
| 1 | Contemporaneous | feelfat | 0.92 |  |  |
|  |  | fearweight | 0.83 |  |  |
|  |  | worthweight | 0.59 |  |  |
|  |  | skipmealsurge | 0.10 |  |  |
|  |  | desirelive | 0.32 |  |  |
|  |  | fearofdeath | 0.17 |  |  |
|  |  | reasonstodie | 0.22 |  |  |
|  |  | passiveideation1 | 0.73 |  |  |
|  | Temporal | feelfat |  | 0.40 | 0.39 |
|  |  | fearweight |  | 0.32 | 0.15 |
|  |  | worthweight |  | 0.44 | 0.24 |
|  |  | skipmealsurge |  | 0.20 | 1.10 |
|  |  | desirelive |  | 0.43 | 0.20 |
|  |  | fearofdeath |  | 0.25 | 0.10 |
|  |  | reasonstodie |  | 0.38 | 0.15 |
|  |  | passiveideation1 |  | 0.08 | 0.19 |
|  |  |  |  |  |  |
| 2 | Contemporaneous | worthweight | 0.18 |  |  |
|  |  | foodrulesrestrict | 0.22 |  |  |
|  |  | feelfat | 0.00 |  |  |
|  |  | restrictfoodurge | 0.12 |  |  |
|  |  | fearofdeath | 0.20 |  |  |
|  |  | desirelive | 0.11 |  |  |
|  |  | passiveideation1 | 0.12 |  |  |
|  |  | reasonstodie | 0.11 |  |  |
|  | Temporal | worthweight |  | 0.37 | 0.24 |
|  |  | foodrulesrestrict |  | 0.01 | 0.85 |
|  |  | feelfat |  | 0.43 | 0.00 |
|  |  | restrictfoodurge |  | 0.23 | 0.05 |
|  |  | fearofdeath |  | 0.53 | 0.10 |
|  |  | desirelive |  | 0.68 | 0.66 |
|  |  | passiveideation1 |  | 0.46 | 0.71 |
|  |  | reasonstodie |  | 0.35 | 0.45 |
|  |  |  |  |  |  |
| 3 | Contemporaneous | feelfat | 0.93 |  |  |
|  |  | fearweight | 0.52 |  |  |
|  |  | mirrorchecking | 1.07 |  |  |
|  |  | restrictfoodurge | 0.23 |  |  |
|  |  | desirelive | 0.67 |  |  |
|  |  | reasonstodie | 0.62 |  |  |
|  |  | passiveideation1 | 0.37 |  |  |
|  |  | problemsolving | 0.34 |  |  |
|  | Temporal | feelfat |  | 0.05 | 0.43 |
|  |  | fearweight |  | 0.26 | 0.51 |
|  |  | mirrorchecking |  | 0.20 | 0.05 |
|  |  | restrictfoodurge |  | 0.18 | 0.46 |
|  |  | desirelive |  | 0.35 | 0.43 |
|  |  | reasonstodie |  | 0.87 | 0.47 |
|  |  | passiveideation1 |  | 0.80 | 0.39 |
|  |  | problemsolving |  | 0.44 | 0.41 |

**Supplementary Table 2**

Bridge Centrality for Individual Contemporaneous and Temporal Networks

| Patient | Network | Node | Strength | InStrength | OutStrength |
| --- | --- | --- | --- | --- | --- |
| 1 | Contemporaneous | feelfat | 0.06 |  |  |
|  |  | fearweight | 0.02 |  |  |
|  |  | worthweight | 0.00 |  |  |
|  |  | skipmealsurge | 0.10 |  |  |
|  |  | desirelive | 0.03 |  |  |
|  |  | fearofdeath | 0.00 |  |  |
|  |  | reasonstodie | 0.00 |  |  |
|  |  | passiveideation1 | 0.15 |  |  |
|  | Temporal | feelfat |  | 0.09 | 0.14 |
|  |  | fearweight |  | 0.00 | 0.00 |
|  |  | worthweight |  | 0.20 | 0.18 |
|  |  | skipmealsurge |  | 0.00 | 0.49 |
|  |  | desirelive |  | 0.28 | 0.12 |
|  |  | fearofdeath |  | 0.14 | 0.10 |
|  |  | reasonstodie |  | 0.38 | 0.08 |
|  |  | passiveideation1 |  | 0.00 | 0.00 |
|  |  |  |  |  |  |
| 2 | Contemporaneous | worthweight | 0.12 |  |  |
|  |  | foodrulesrestrict | 0.13 |  |  |
|  |  | feelfat | 0.00 |  |  |
|  |  | restrictfoodurge | 0.10 |  |  |
|  |  | fearofdeath | 0.20 |  |  |
|  |  | desirelive | 0.10 |  |  |
|  |  | passiveideation1 | 0.02 |  |  |
|  |  | reasonstodie | 0.03 |  |  |
|  | Temporal | worthweight |  | 0.15 | 0.24 |
|  |  | foodrulesrestrict |  | 0.01 | 0.35 |
|  |  | feelfat |  | 0.27 | 0.00 |
|  |  | restrictfoodurge |  | 0.11 | 0.05 |
|  |  | fearofdeath |  | 0.40 | 0.07 |
|  |  | desirelive |  | 0.13 | 0.00 |
|  |  | passiveideation1 |  | 0.05 | 0.25 |
|  |  | reasonstodie |  | 0.05 | 0.22 |
|  |  |  |  |  |  |
| 3 | Contemporaneous | feelfat | 0.39 |  |  |
|  |  | fearweight | 0.16 |  |  |
|  |  | mirrorchecking | 0.31 |  |  |
|  |  | restrictfoodurge | 0.08 |  |  |
|  |  | desirelive | 0.64 |  |  |
|  |  | reasonstodie | 0.12 |  |  |
|  |  | passiveideation1 | 0.04 |  |  |
|  |  | problemsolving | 0.15 |  |  |
|  | Temporal | feelfat |  | 0.03 | 0.25 |
|  |  | fearweight |  | 0.21 | 0.30 |
|  |  | mirrorchecking |  | 0.00 | 0.00 |
|  |  | restrictfoodurge |  | 0.00 | 0.46 |
|  |  | desirelive |  | 0.09 | 0.00 |
|  |  | reasonstodie |  | 0.66 | 0.19 |
|  |  | passiveideation1 |  | 0.10 | 0.03 |
|  |  | problemsolving |  | 0.15 | 0.02 |
